# DNA methylation differences at growth related genes correlate with birth weight: a molecular signature linked to developmental origins of adult disease?

**DOI:** 10.1186/1755-8794-5-10

**Published:** 2012-04-12

**Authors:** Nahid Turan, Mohamed F Ghalwash, Sunita Katari, Christos Coutifaris, Zoran Obradovic, Carmen Sapienza

**Affiliations:** 1 Fels Institute for Cancer Research and Molecular Biology, Temple University School of Medicine, Philadelphia, PA 19140, USA; 2 Center for Information Science and Technology, Temple University, Philadelphia, PA 19122, USA; 3 Department of Obstetrics & Gynecology, University of Pennsylvania School of Medicine, Philadelphia, PA 19104, USA; 4 Department of Pathology and Laboratory Medicine, Temple University School of Medicine, Philadelphia, PA 19140, USA

## Abstract

**Background:**

Infant birth weight is a complex quantitative trait associated with both neonatal and long-term health outcomes. Numerous studies have been published in which candidate genes (*IGF1, IGF2, IGF2R, IGF *binding proteins, *PHLDA2 *and *PLAGL1*) have been associated with birth weight, but these studies are difficult to reproduce in man and large cohort studies are needed due to the large inter individual variance in transcription levels. Also, very little of the trait variance is explained. We decided to identify additional candidates without regard for what is known about the genes. We hypothesize that DNA methylation differences between individuals can serve as markers of gene "expression potential" at growth related genes throughout development and that these differences may correlate with birth weight better than single time point measures of gene expression.

**Methods:**

We performed DNA methylation and transcript profiling on cord blood and placenta from newborns. We then used novel computational approaches to identify genes correlated with birth weight.

**Results:**

We identified 23 genes whose methylation levels explain 70-87% of the variance in birth weight. Six of these (*ANGPT4, APOE, CDK2, GRB10, OSBPL5 *and *REG1B*) are associated with growth phenotypes in human or mouse models. Gene expression profiling explained a much smaller fraction of variance in birth weight than did DNA methylation. We further show that two genes, the transcriptional repressor *MSX1 *and the growth factor receptor adaptor protein *GRB10*, are correlated with transcriptional control of at least seven genes reported to be involved in fetal or placental growth, suggesting that we have identified important networks in growth control. *GRB10 *methylation is also correlated with genes involved in reactive oxygen species signaling, stress signaling and oxygen sensing and more recent data implicate *GRB10 *in insulin signaling.

**Conclusions:**

Single time point measurements of gene expression may reflect many factors unrelated to birth weight, while inter-individual differences in DNA methylation may represent a "molecular fossil record" of differences in birth weight-related gene expression. Finding these "unexpected" pathways may tell us something about the long-term association between low birth weight and adult disease, as well as which genes may be susceptible to environmental effects. These findings increase our understanding of the molecular mechanisms involved in human development and disease progression.

## Background

One common non-disease phenotype that puts children at increased risk for multiple adverse outcomes is "low birth weight". Low birth weight is simply the transformation of the quantitative phenotype of birth weight into a discrete trait by truncation at the lowest decile of infant birth weights; *i.e*., a birth weight of less than 2,500 g. Low birth weight increases the risk of neonatal death by four-fold in comparison with infants weighing 2,500-2,999 g and by 10-fold in comparison with infants weighing 3,000-3,499 g [1]. This increased risk continues after birth [1]. The financial cost of low birth weight is also substantial. In the United States, low birth weight babies account for 47% of the cost of all infant hospitalizations and 42% of these costs are borne by Medicaid [2]. The long-term costs continue to accumulate throughout life because low birth weight is associated with cognitive impairment [3] and increased risk of childhood and adult diseases, including obesity, hypertension, cardiovascular disease and type II diabetes [4-7].

Epidemiological studies have also shown a 2.6× increased risk of low birth weight in children conceived using assisted reproduction techniques (ART) such as *in vitro *fertilization (IVF) [8]. In 2009, ART resulted in 60,190 infants, contributing to > 1% of annual births in the United States [9]. To date there have been over 3.75 million ART births worldwide [10], and as the oldest of these children are only now entering their 30's, there is concern regarding any long-term health effects associated with low birth weight in this population.

The mechanisms linking low birth weight to adverse long-term health outcomes are not well understood but may be related to defective placentation [11-13], abnormal programming of metabolic pathways, including glucose utilization [4,14] and restrictions in the size of stem cell populations that lead to reduced organ size and function [15,16]. The overall lack of direct information concerning the mechanisms by which low birth weight is coupled to childhood and adult diseases provides a compelling reason for defining the factors that affect birth weight.

Numerous studies have been published in which the expression of genes known to affect growth have been surveyed with respect to birth weight, including insulin-like growth factor 1 (*IGF1*), *IGF2, IGF2 *receptor *(IGF2R), IGF *binding proteins, pleckstrin homology-like domain family A, member 2 (*PHLDA2*) and pleiomorphic adenoma gene-like 1 (*PLAGL1*) [13,17-26]. However, few of the associations have been replicated in independent populations and very little of the trait variance is explained by these measures. For example, we failed to find significant correlation between infant birth weight and transcript levels of IGF2, IGF2R or the ratio of IGF2/IGF2R transcripts in cord blood and placenta from newborns, measured at delivery [27].

Birth weight is a complex phenotype that represents the sum of many processes and gene expression patterns operating throughout embryonic and fetal development. It is, perhaps, not surprising that a strong association between birth weight and the expression of any particular gene, measured at a single time point (delivery, in most cases), has proven elusive, even for genes which have mechanistic links to growth. It is possible that the mechanism-based candidates are, indeed, the genes that are most relevant to birth weight but that the expression of these genes at delivery is not the appropriate measure of their action. Alternatively, it is possible that the activities of other genes, yet to be defined, are more predictive of birth weight than the current candidates.

The failure of mechanism-based candidate gene transcript approaches to explain a substantial fraction of birth weight trait variance (*e.g. *[27]) prompted us to consider a more agnostic approach. In the present study, we have used gene promoter-specific DNA methylation levels as a quantitative measure of "expression potential" to identify additional candidate genes. We chose this measure because at least 50% of human genes show an inverse correlation between promoter DNA methylation levels and gene expression [28,29]. We combined DNA methylation profiling with a novel "machine learning" approach to identify additional candidate genes that are correlated with birth weight. We also evaluated whether DNA methylation levels of a suite of mechanism-based candidates explains birth weight trait variance better than transcript level of the same genes.

## Methods

### Ethics statement and samples

Written, informed consent was obtained in advance from the mother of each newborn (University of Pennsylvania I.R.B. approved protocol no. 804530).

We have provided the demographic data showing maternal age, race, parity, fetal sex, gestational age, birth weight (at delivery) and birth weight percentiles for the individuals in the GoldenGate and Infinium Methylation Assays in an additional file (Additional file 1).

### Sample collection and processing

Cord blood and placenta samples were collected from each newborn. All cord blood samples were collected within 20 minutes of delivery. The umbilical cord was wiped with sterile saline solution to minimize maternal blood contamination and the cord vein was punctured with a 21 G needle. Whole cord blood (6-10 ml) was collected in an EDTA-Vacutainer tube. An aliquot (3 ml) of cord blood was transferred to a 15 ml Falcon tube containing RNALater RNA Stabilization Reagent (Ambion, USA), following the manufacturers guidelines, to stabilize the RNA. The remaining cord blood was saved for DNA extraction. All cord blood DNA and RNA samples were initially stored at 4°C, and nucleic acid extractions were performed within 2-4 days of collection.

Tissue samples were collected and processed within five hours of delivery [30]. Placental tissue (1.5-2.5 cm^3^) was excised from the fetal surface of the placenta, directly behind the cord insertion site. The sample was rinsed extensively with sterile saline solution to minimize maternal blood contamination. Half of the tissue sample was sectioned into smaller pieces (0.5 cm^3^), transferred to a 15 ml Falcon tube and immersed in RNALater RNA Stabilization Reagent (Ambion, USA), following the manufacturers guidelines. The remaining tissue was transferred to a 15 ml Falcon tube for DNA extraction. All tissue DNA and RNA samples were initially stored at 4°C, and nucleic acid extractions were performed within 2-4 days of collection. Approximately 4-5 mg of tissue was used to extract genomic DNA and RNA. The remaining tissue was stored at -80°C.

### DNA and RNA isolation

Cord blood DNA was isolated using the Archive Pure DNA Blood Kit (Fisher Scientific Company, USA), following the manufacturers guidelines. Placenta genomic DNA was extracted using standard phenol-chloroform extraction methods. The isolated DNA was resuspended in TrisCl (10 mM, pH 8.0) and stored at -80°C until further use. Cord blood RNA was isolated using the PerfectPure RNA Blood Kit (Fisher Scientific Company, USA), following the manufacturers guidelines. Placenta total cellular RNA was extracted using TRIzol^® ^Reagent (Invitrogen Corporation, USA), following the manufacturers guidelines. The isolated RNA was resuspended in Milli-Q water and stored at -80°C until further use. Isolated DNA and RNA were analyzed by agarose gel electrophoresis and quantified using a NanoDrop ND1000 (Thermo Fisher Scientific, USA). RNA samples were further assessed for quality using the Agilent 2100 Bioanalyzer (Santa Clara, USA) prior to the whole genome expression analysis.

### Transcriptome profiling

Whole genome expression was analyzed in cord blood and placenta RNA template for 48 individuals using Illumina's HumanHT-12 v3 Expression BeadChip (Illumina, USA), which provides coverage for more than 47,000 transcripts and known splice variants across the human transcriptome. Isolated total RNA was quantified using a NanoDrop ND1000 (Thermo Fisher Scientific, USA) and assessed for quality using the Agilent 2100 Bioanalyzer (Santa Clara, USA) prior to the whole genome expression analysis. By Illumina criteria, RNA samples for gene expression array analysis were required to have a RIN > 7, an OD 260:280 of 1.9-2.0, an OD 260/230 of > 1.8 and a 28S:18S ratio of the ribosomal bands of > 1.5. Expression profiling was accomplished using the HumanHT-12 v3 whole-genome gene expression direct hybridization assay (Illumina, USA), following the manufacturers guidelines. Illumina's Total Prep RNA Amplification Kit (Ambion, USA) was used to transcribe 200 ng total RNA to cDNA, followed by an in vitro transcription step to generate labeled cRNA, following the manufacturers guidelines. The labeled probes were then mixed with hybridization reagents and hybridized at 58°C for 16 h to the Bead Chips. The Bead Chips were washed and stained, as per the manufacturer's instructions, and then scanned using the Illumina Bead Array Reader. The Bead Scan Software (Illumina, USA) was used to measure fluorescence intensity at each probe, which corresponds to the quantity of the respective mRNA in the original sample. Illumina's GenomeStudio Gene Expression Module v1.0 was used to analyze the data. Briefly, raw intensity data was corrected by background subtraction in the Genome Studio module and normalized using the Quantile normalization algorithm.

### Quantitative real time RT-PCR

First-strand cDNA was obtained using Superscript™ III Reverse Transcriptase (RT) (Invitrogen Corporation, USA). To produce cDNA from total RNA, a mixture containing 1 μg extracted total RNA, 0.5 μg oligo(dT)18 primer and 1 μl dNTP mix (10 mM each base) in final 13 μl of solution was heated to 65°C for 5 min, cooled down on ice for 2 min, and then added to a 7 μl of reaction mixture (4 μl Superscript™ III RT buffer (10×), 1 μl DTT (0.1 M), 1 μl RNaseOUT™ Recombinant RNase inhibitor (40 U/μl; Invitrogen Corporation, USA) and 1 μl Superscript™ III M-MLV reverse transcriptase (200 U/μl), for reverse transcription at 50°C for 60 min. Reactions were terminated at 70°C for 15 min. RT products were stored at -20°C until use. Quantitative real time RT-PCR assays were carried out using a 7700 Sequence Detector (Applied Biosystems, USA). All probes spanned exon/intron boundaries to prevent genomic DNA amplification.

Steady state mRNA levels of IGF2BP2, IGFBP1, IGFBP2, IGFBP3, PLAGL1 and housekeeping genes GAPDH and TBP were measured using gene-specific TaqMan probes (Applied Biosystems, USA, product numbers: Hs01118009_m1, Hs00236877_m1, Hs01040719_m1, Hs00426289_m1, HS00414677_m1, HS02758991_G1 and HS00920497_M1, respectively). Taqman PCR reactions were performed by mixing 1 μl of cDNA (50 ng/μl) with 19 μl of reaction mixture (10 μl Taqman Master Mix (2×), 1 μl Taqman primer (20×), and 8 μl nuclease free dH_2_O) and amplified under the following conditions: 50°C for 2 min, 95°C for 10 min, followed by 45 cycles of 95°C for 15 s and 60°C for 60 s.

Steady state mRNA levels of IGF2, IGF2R and housekeeping gene GAPDH were measured using gene-specific primers (IGF2 forward 5'-TCTGACCTCCGTGCCTA-3', IGF2 reverse 5'-TTGGGATTGCAAGCGTTA-3', IGF2R forward 5'-ACCTCAGCCGTGTGTCCTCT-3', IGF2R reverse 5'-CTCCTCTCCTTCTTGTAGAGCAA-3', GAPDH forward 5'-GAGTCAACGGATTTGGTCGT-3' and GAPDH reverse 5'-TTGATTTTGGAGGGATCTCG-3') and QuantiFast SYBR Green PCR Master Mix (Qiagen, USA). PCR reactions were performed by mixing 1 μl of cDNA (50 ng/μl) with 24 μl of reaction mixture (10 μl QuantiFast SYBR Green PCR Master Mix (2×), 2.5 μl forward primer (10 μM), 2.5 μl reverse primer (10 μM), and 6.5 μl nuclease free dH_2_O) and amplified under the following conditions: 95°C for 5 min, followed by 45 cycles of 95°C for 10 s and 60°C for 30 s. A melting curve analysis of the PCR products was performed to verify their specificity and identity. Relative gene expression levels were obtained using the ΔΔCt method [31].

### Bisulfite conversion

Unmethylated cytosine in genomic DNA (0.5-1 μg) was converted to uracil by treatment with sodium bisulfite using the EZ DNA Methylation Kit™ (Zymo Research Corp., USA), following the manufacturers guidelines. The bisulfite-converted DNA was resuspended in 20 μl TrisCl (10 mM, pH 8.0) buffer and stored at -20°C until further use. All converted DNA samples were used within one month of the bisulfite conversion.

### GoldenGate methylation assay

Site-specific CpG methylation was analyzed in the bisulfite converted cord blood and placenta DNA template for 22 individuals, in duplicate, using a custom-designed methylation bead array platform, following the manufacturers guidelines (Illumina, USA) and as previously described [32]. The GoldenGate methylation array contained probes for 1,536 CpG dinucleotides located in the promoters of more than 700 genes (Illumina Inc., USA) [33,34]. In addition, the array includes CpGs for all known human imprinted genes. Illumina's GenomeStudio Methylation Module v1.0 was used to analyze the data and assign site-specific DNA methylation β-values to each CpG site. The extent of methylation (β-value) at each CpG site was determined by comparing the proportion of signal from methylated and unmethylated alleles in the DNA sample.

### Infinium methylation assay

Site-specific CpG methylation was analyzed in the bisulfite converted cord blood and placenta DNA template for 48 individuals using Illumina's HumanMethylation27 BeadChip array, following the manufacturers guidelines (Illumina, USA). The array contained probes for 27,578 CpG dinucleotides located in the proximal promoter regions of over 14,000 consensus coding sequences (CCDS) genes throughout the genome. In addition, the array included 110 miRNA promoters and imprinted genes. Four bead chips were used for each tissue type, and these were processed simultaneously. Briefly, 1 μg of bisulfite converted DNA was isothermally amplified at 37°C overnight. The amplified DNA product was fragmented by an endpoint enzymatic process and the fragmented DNA was precipitated, resuspended and applied to the array and hybridized overnight. A single-base extension reaction was carried out and the fluorescently stained chip was imaged using the Illumina Bead Array Reader and the Bead Scan Software (Illumina, USA). The assay contained controls to assess the following parameters: staining, hybridization, target removal, extension, bisulfite conversion, G/T mismatch, as well as negative controls and non-polymorphic controls. The experiments passed all quality controls successfully (Please see Illumina's "GenomeStudio Methylation Module User Guide" manual for greater details regarding the criteria used to assess the controls). Illumina's GenomeStudio Methylation Module v1.0 was used to analyze the data to assign site-specific DNA methylation β-values to each CpG site. The extent of methylation (β-value) at each CpG site was determined by comparing the proportion of signal from methylated and unmethylated alleles in the DNA sample.

### Pyrosequencing methylation assay

Site-specific CpG methylation was analyzed in the bisulfite converted cord blood DNA template for *PRSS21*, and in the placenta DNA template for *ANGPT4, PGRMC1 *and *RGS14*, using custom designed bisulfite pyrosequencing assays (Qiagen, USA). The assays were designed to target the same CpGs interrogated by the GoldenGate and Infinium arrays. Briefly, 500 ng bisulfite converted DNA was used for generating PCR amplified templates for pyrosequencing. The primer sequences are following: *ANGPT4 *forward (5' GGGTTGAATGGATTTTTGTTGGATGAATG 3'), reverse (5' CCTTCCCTAAACACAAAAAACTATCTCT 3') and sequencing (5' ACTAACAACCTAACTCTT 3'); *PGRMC1 *forward (5' TGTTTGGTGATTGAGTAAATTAGTAATTGT 3'), reverse (5' TCCTTAATAACCCTTCCCCAATTC 3') and sequencing (5' GTTGTGTATTGATTTTAGTAATTT 3'); *PRSS21 *forward (5' GGGTTTGGGTTATATTAAGAAGTGT 3'), reverse (5' TTCACCCTCCTAAACCCAAAAACTATT 3') and sequencing (5' AGTGTGGTTGAAGAT 3'); *RGS14 *forward (5' GGGTAGGTAGTGGAGAGAGT 3'), reverse (5' CTCTCTTAAACCTTACTTCTTTCTATAATT 3') and sequencing (5' GTGGAGAGAGTTTGAT 3'). For *ANGPT4 *the 5'-biotin modification is on the forward primer, whereas for *PGRMC1, PRSS21 *and *RGS14 *the 5'-biotin modification is on the reverse primer.

The PCR reaction (30 μl) was following: 25 ng of bisulfite DNA, 0.75 U HotStar Taq Polymerase (Qiagen, USA), 1× PCR buffer, 3 mM MgCl_2_, 200 μM of each dNTP, and 6 pmol of each forward and reverse primer. Recommended PCR cycling conditions were: 95°C for 15 min; 45 cycles (95°C for 30 s; 60°C for 30 s; 72°C for 30 s); 72°C for 5 min. The biotinylated PCR product (10 μl) was used for each assay with 1× the respective sequencing primer. Pyrosequencing was done using the PSQ96HS system using the PyroMark Gold Reagent Kit, following the manufacturers guidelines (Qiagen, USA). Methylation was quantified using PyroMark Q-CpG Software (Qiagen, USA), which calculates the ratio of converted C's (T's) to unconverted C's at each CpG and expresses this as a percentage methylation.

### Regression analyses methodology

In order to have a reliable and meaningful comparison of gene expression and DNA methylation levels, the values were balanced by a min-max normalization procedure which transformed them to (0,1) range [35]. After normalization, the L_1_-reqularized linear regression procedure [36] was applied to identify candidate genes associated with birth weight. L_1_-regularized regression outperforms Ridge regression [37] and L2-regression [38], and enforces removing outliers and irrelevant genes, focusing on a small number of relevant genes [39-41]. The procedure was applied to two groups of DNA methylations with different numbers of CpG sites and gene expressions, which are referred to as "predictors" hereafter. Finally, the bootstrap method was used [42] to assess the significance of the models selected by the L_1_-regularized regression procedure.

### L_1_-regularized regression

Assuming one is given *n *samples *S *= (*X*_1_, *y*_1_), ..., (*X_n_, y_n_*) where each sample consists of *k *real-valued predictors *X_i _∈ R^k ^*which represent array signal intensities, and a real valued dependent variable *y_i _*which represents the birth weight percentiles. The problem was to find the effect of those predictors *X_i _*on the dependent variable *y_i_*. L_1_-regularized regression accomplished this by finding a coefficient vector β that minimizes

$$\sum_{i=1}^{n}\;{\left(y_{i}-f\left(X_{i}\right)\right)}^{2}+\lambda \sum_{j=0}^{k}\;∥\beta ∥$$

where

$$f\left(X_{i}\right)=\beta_{0}+\sum_{j=1}^{k}\;\beta_{j}X_{ij}+\varepsilon$$

Here, ε is the error induced by the model and/or noise in the data which is independent of the birth weight, and *λ *controls the tradeoff between fitting the data and having a small number of parameters.

### Two-stage L_1_-regularized regression

In the first stage of this process, L_1_-regularized regression was applied to eliminate irrelevant predictors while keeping a small number of relevant predictors. Since regression models usually suffer from over fitting when applied to small sample sizes, a leave-one-out cross validation (LOOCV) was used to assess the model. In this process, one sample was excluded while the regression model was trained on the remaining samples. The performance of the trained model was then evaluated on the hold-out sample. This process was repeated *n *times where each time, a different sample was held out for testing. After applying L_1_-regularized regression *n *times, the number of times each predictor appeared in all *n *cross validation experiments was counted. A predictor was called *m*-stable if it appeared in *m *cross validations. All *m*-stable predictors for the *m*-model were selected; the value of the *m *was determined later. The *m*-model was called stable if L_1_-regularized regression was applied on *h *predictors and the final *m*-model contained all *h *predictors. If the *m*-model was not stable, the LOOCV process was repeated on the predictors in the *m*-model several times, until a stable model was achieved. The stable *m*-model was a linear combination of a subset of the original predictors. However, a linear combination of predictors might not express the response variable very well. Therefore, the second stage effects were explored by analyzing all pair wise interactions among candidate stable predictors selected in the first stage. A new set of predictors was generated which contained the predictors in the *m*-model, as well as all pair wise interactions between the predictors in the *m*-model. The same process as in the first stage was applied to get a stable model, which explored not only the marginal effects of the predictors but also the joint interaction effects between those predictors. Given *n *samples, an application of the proposed two-stage L_1_-regularized regression process *n *times resulted in *n m*-models, where *m *= 1,.., *n*.

### Choosing the best model

To test the accuracy of the model, we computed the adjusted R^2^, which is a modification of R^2 ^that adjusts for the number of explanatory terms in a model. Unlike R^2^, the adjusted R^2 ^increases only if the new term improves the model more than would be expected by chance. In other words, the adjusted R^2 ^is the amount of variance in the outcome that the model explains in the population. It was discovered that the model that had the largest adjusted R^2 ^value also had low stability. In order to get a model that was stable as well as accurate, all *n m*-models, starting from the more stable *n *-model, were searched in a greedy fashion, until a model with an adjusted R^2 ^value larger than 0.5 was found, which was called the *k *-model. Then all *h *-models were searched, where *h *= *k*-1,..,1, that had the same predictors as the *k *-model. The aim of this search was to find another model that had the same number of predictors as in the *k *-model, but also achieved a higher adjusted R^2 ^value than the *k *-model. This model had the advantage of being optimized to contain a small number of predictors, while also being stable and accurate.

### Bootstrap method

A popular way of evaluating the reliability of any computational method is using the bootstrap analysis [43,44]. The first step in a bootstrap analysis is to re-sample the set of genes. Then the L_1 _procedure is applied to the re-sampled dataset. The adjusted R^2 ^of the re-sampled dataset represents an estimate of how a different set of genes explain the variance of the birth weight. If the R^2 ^on the re-sampled dataset is similar to or less than the R^2 ^on the whole set of genes computed by the L_1 _procedure, this increases the confidence in the model generated by applying the L_1 _procedure on the whole set of genes. By re-sampling a number of times it is possibly to draw the distribution of the R^2 ^and hence compute the reliability of the L_1 _procedure.

### Statistical analysis

To measure the correlation between expression and methylation genes, Pearson's linear correlation two-tailed test was used, with the hypothesis of no correlation using a Student's *t *distribution for a transformation of the correlation. The null hypothesis of the Pearson's linear correlation was that there is no correlation between the two predictors. The *P *value determined whether the null hypothesis was rejected, or if there was no evidence to reject it. *P*-values 0.01 were considered significant.

### Software

Math works Matlab R2010b software was used to run all the experiments. The glmnet implementation of lasso regression [45,46] was used for generalized linear modeling. This algorithm was based on convex penalties and cyclic coordinate descend, computed along the regularization path, which can handle large problems in reasonable time. The algorithm had an embedding strategy for choosing the best value of lambda which determines the weight of the penalized regularization term.

## Results and discussion

### Mechanism-based candidate gene transcription and birth weight

**Figure 1:**
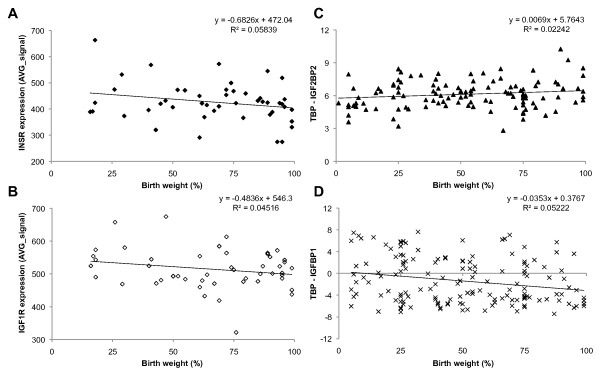
**Expression levels of mechanism-based candidate genes versus birth weight**. Illumina's HumanHT-12 v3 Expression of **(A) **INSR (transcript ID: ILMN_1670918) in cord blood (n = 48), and **(B) **IGF1R (transcript ID: ILMN_1675048) in placenta (n = 48). Real time RT-PCR expression of **(C) **IGF2BP2 in cord blood (n = 119), and **(D) **IGFBP1 in placenta (n = 150).

We measured global transcription patterns in cord blood and placenta of 48 newborns using Illumina's HumanHT-12 v3 Expression BeadChip (see Methods). We also measured transcript levels of selected candidate genes in a larger group of individuals (n = 105-254) by real time RT-PCR. We then performed linear regression of birth weight, corrected for gestational age (birth weight percentile), against cord blood and placenta transcript levels of IGF1, IGF1 receptor (IGF1R), IGF2, IGF2 mRNA binding proteins 1-3 (IGF2BP1-3), IGF2R, IGF binding proteins 1-7 (IGFBP1-7), insulin (INS), INS receptor (INSR), INSR-related receptor (INSRR), PHLDA2 and PLAGL1. We did not observe any strong correlation between birth weight and transcript level of any of these "mechanism-based" candidate genes, with the strongest correlation (R^2 ^= 0.058) found for INSR in cord blood (Table 1). The associations with the best correlations are plotted in Figure 1 to illustrate the strength, or lack thereof, of the associations. Correlation coefficients for all candidate genes are given in Table 1.

**Table 1:** Correlation of mechanism-based candidate gene expression levels with birth weight

Gene Symbol	Transcript ID	HumanHT-12 v3 Expression vs. Birth Weight % (R^2^)		Real Time RT-PCR Expression vs. Birth Weight % (R^2^)	
		Cord Blood (n = 48)	Placenta (n = 48)	Cord Blood	Placenta
**IGF1**	ILMN_2056087	2.0E-04	0.017	nd	nd
	ILMN_1709613	0.003	0.002		
**IGF1R**	ILMN_1675048	0.009	**0.045**	nd	nd
**IGF2**	ILMN_1699867	1.3E-05	0.004	1.2E-04 (n = 190)	4.5E-04 (n = 254)
	ILMN_2298035	0.008	0.001		
	ILMN_2413956	0.003	0.003		
**IGF2BP1**	ILMN_1733807	0.007	1.0E-04	nd	nd
**IGF2BP2**	ILMN_1702447	0.003	0.016	**0.022 (n = 119)**	1.0E-07 (n = 114)
**IGF2BP3**	ILMN_1807423	0.056	0.007	nd	nd
**IGF2R**	ILMN_1807662	0.006	3.0E-04	0.005 (n = 194)	6.1E-04 (n = 241)
**IGFBP1**	ILMN_2387385	0.014	0.001	ne	**0.052 (n = 150)**
	ILMN_1728445	0.001	0.001		
**IGFBP2**	ILMN_1725193	0.006	0.031	ne	0.003 (n = 110)
**IGFBP3**	ILMN_1746085	0.007	0.002	ne	0.001 (n = 135)
	ILMN_2396875	0.009	0.002		
**IGFBP4**	ILMN_1665865	0.006	0.003	nd	nd
**IGFBP5**	ILMN_2132982	0.014	0.002	nd	nd
	ILMN_1750324	0.001	0.003		
**IGFBP6**	ILMN_1669362	0.001	0.009	nd	nd
**IGFBP7**	ILMN_2062468	2.5E-05	0.005	nd	nd
**INS**	ILMN_1666966	0.022	0.034	nd	nd
**INSR**	ILMN_1670918	**0.058**	0.031	nd	nd
**INSRR**	ILMN_1715374	0.007	3.9E-05	nd	nd
**PHLDA2**	ILMN_1671557	0.036	0.001	nd	nd
**PLAGL1**	ILMN_1815121	0.001	0.009	0.006 (n = 105)	0.013 (n = 136)
	ILMN_2356955	0.014	0.004		
IGF2/IGF2R*		n/a	n/a	0.002 (n = 186)	0.002 (n = 241)

Multiple entries represent data for multiple transcripts on the array. The best correlation obtained in each group is shown in bold

* Ratio IGF2/IGF2R expression

n/a = not applicable

nd = not done

ne = not expressed

We also used L_1 _regularized regression ([36,39-41] and see Methods) to evaluate the contribution of transcript levels of these 19 growth-related genes, collectively, to explain birth weight trait variance. This analysis was performed using the transcript levels and birth weights of the 48 individuals profiled on the whole transcriptome array. L_1 _regression analysis is a machine-learning approach that seeks to identify features relevant to a particular phenotype from amongst a large background of irrelevant features (although the relevant features in the present experiment were defined as transcript levels of the 19 mechanism-based candidates). It evaluates the strength of association for each feature (transcript) by performing successive "leave one sample out" experiments and determines how many of the resample data sets exhibit non-zero correlations between transcript level and birth weight. A threshold of 45/48 (94%) non-zero correlations was adopted for this analysis. The 19-gene mechanism-based candidate model (using all of the genes in Table 1) resulted in an adjusted R^2 ^of 0.24. Although this is a significant improvement over the birth weight trait variance explained by any individual gene, it still leaves more than 75% of the trait variance unexplained.

### Evaluation of DNA methylation differences in mechanism-based candidates

We then evaluated whether promoter DNA methylation levels of the mechanism-based candidate genes would perform better than single time-point transcript level to explain birth weight trait variance in two methylation profiling experiments. In the first experiment, we measured DNA methylation levels at 1,536 CpG sites in cord blood and placenta of 22 individuals using a custom-designed DNA methylation array (which uses the "GoldenGate" assay to measure methylation levels; Illumina, Inc. USA, see Methods and [32]). The 1,536 CpG sites examined were located in 740 loci that were selected for functions in cell growth, proliferation or embryonic development [32]. CpGs in 16 of the mechanism-based candidate genes were included on the array, as well as probes for the *IGF2/H19 *DMR (the array did not contain probes for *IGF2BP1, IGF2BP2 *or *INSRR*). In the second experiment, methylation levels at 27,578 CpGs in 14,495 genes were assayed (using an Illumina Infinium array; Illumina, Inc. USA) in the same 48 individuals for whom transcription was evaluated in Table 1. CpGs in 17 of the mechanism-based candidate genes were included on the array (the array did not contain probes for *INSR *or *INSRR*). We did not observe a strong correlation between birth weight and methylation level of any of these "mechanism-based" candidate genes (Table 2), with the strongest correlation (R^2 ^= 0.163) found for *PHLDA2 *methylation levels in placenta on the GoldenGate array (Table 2).

**Table 2:** Correlation of mechanism-based candidate gene methylation levels with birth weight

Gene Symbol	GoldenGate CpG ID	GoldenGate Methylation vs. Birth Weight % (R^2^)		Infinium CpG ID	Infinium Methylation vs. Birth Weight % (R^2^)	
		Cord Blood (n = 22)	Placenta (n = 23)		Cord Blood (n = 48)	Placenta (n = 48)
***IGF1***	cg17084217	0.004	0.004	cg01305421	0.005	0.007
	cg25163611	1.0E-04	0.031	cg14568338		
***IGF1R***	cg19714640	**0.097**	0.038	cg22375192	0.011	0.021
	cg20742855	0.005	0.018	cg02166532	0.006	0.001
***IGF2***	cg10649864	0.007	0.077	cg02807948	0.049	4.0E-04
	cg17626526	0.040	0.026	cg13756879	4.0E-04	0.001
	cg17084217	0.011	3.0E-04	cg20339650	0.014	4.0E-04
				cg22956483	3.0E-04	0.001
				cg01305421	0.032	0.003
***IGF2BP1***		n/a	n/a	cg06638433	0.005	0.044
				cg13877465	0.019	8.3E-05
***IGF2BP2***		n/a	n/a	cg18234011	0.005	0.024
				cg24450631	0.005	0.006
***IGF2BP3***	cg00508334	3.5E-05	0.028	cg02860543	0.049	1.2E-05
	cg21413760	0.062	3.1E-07	cg19042950	1.2E-05	0.002
***IGF2R***	cg07148501	0.009	0.076	cg00230368	0.007	0.014
	cg12721534	0.014	0.063	cg14556618	8.4E-05	1.0E-04
***IGFBP1***	cg20666158	0.015	0.059	cg05660795	0.033	0.014
	cg23864854	0.048	0.028	cg27447599	0.021	0.018
***IGFBP2***	cg07828219	0.032	0.018	cg25854162	0.004	0.011
	cg17207942	0.035	0.001	cg26187237	6.7E-05	0.015
***IGFBP3***	cg12826145	0.023	0.012	cg04796162	0.014	0.036
	cg14625938	0.001	0.010	cg06713098	0.027	0.002
				cg08831744	0.001	0.003
				cg15898840	0.026	0.003
				cg22083798	0.029	0.042
***IGFBP4***	cg03940014	0.054	0.008	cg00512374	0.008	0.022
	cg22392383	0.018	0.042			
***IGFBP5***	cg20419545	0.066	0.001	cg19008649	0.021	0.005
	cg24617085	0.067	0.017	cg22467567	0.001	0.006
***IGFBP6***	cg00122038	0.009	0.011	cg01773854	0.051	1.0E-04
	cg22732012	0.072	2.0E-04	cg08629913	0.024	0.003
***IGFBP7***	cg00431950	0.023	0.037	cg00884221	0.002	0.001
	cg16546204	0.026	0.014	cg03876618	3.3E-05	0.001
***INS***	cg13349859	0.001	0.020	cg00613255	0.001	0.005
	cg14426263	0.008	0.005	cg03366382	1.0E-04	0.044
				cg13993218	0.003	0.012
				cg25336198	0.005	0.008
***INSR***	cg05427477	0.002	0.084	cg01263716	n/a	n/a
	cg19110381	0.072	0.001	cg01505590		
***PHLDA2***	cg03637064	0.019	**0.163**	cg04720330	4.0E-05	0.062
	cg18242686	0.024	0.006	cg11961618	0.039	0.014
				cg14415214	0.001	**0.081**
				cg21259253	4.0E-04	0.031
				cg26799802	3.0E-04	0.035
				cg00702231	0.019	0.031
				cg07077459	**0.055**	0.006
***PLAGL1***	cg10923987	0.002	0.052	cg08263357	3.8E-06	0.006
	cg12757684	0.067	0.062	cg12757684	0.001	0.013
				cg14161241	0.002	0.030
				cg17895149	0.001	0.009
				cg22378065	0.017	0.034
				cg25350411	0.002	0.001
				cg00613255	0.007	0.003
				cg03366382	0.010	0.001
IGF2/H19*	cg25871270	0.001	0.065		n/a	n/a
	cg19731870	0.002	0.008			

Multiple entries represent data from multiple CpG sites. The best correlation obtained in each group is shown in bold

* IGF2/H19 differentially methylated region (DMR)

n/a = not applicable i.e. no probes on array

We then used the same L_1 _regularized regression method used to evaluate the contribution of transcript level to birth weight trait variance, above. Methylation levels at these genes explained 26% of birth weight trait variance in the first data set and 46% of trait variance in the second data set, suggesting that promoter methylation levels are at least as good, and possibly better, at explaining birth weight trait variance than transcript level.

### Identification of additional candidate genes through machine-learning

The great strength of L_1 _regularized regression is the *de novo *identification of relevant features among a large background of irrelevant features. In the second phase of the analysis, it evaluates each relevant feature, singly and in combination with each other, for non-zero contributions to trait variance. We performed L_1 _regression on promoter methylation levels of the 740 genes in the 22 individual data set used to evaluate the mechanism-based candidate genes, above, to determine which of the genes, singly or in combination, contributed the largest fraction to birth weight trait variance.

This approach identified six genes (*APOE, MSX1, GRB10, PGRMC1, RGS14 *and *SHMT2*), whose methylation level in cord blood and/or placenta accounted for 78% of the variance in birth weight, which is substantially higher than the fraction of trait variance explained by the 19 mechanism-based candidates (26%). We note that at least two of the candidate genes have been linked to growth related phenotypes. *APOE *has been associated with body mass index (BMI) [47,48] and bone density [49] in humans and *Grb10 *has been linked to both placental and fetal growth in the mouse [50,51].

**Figure 2:**
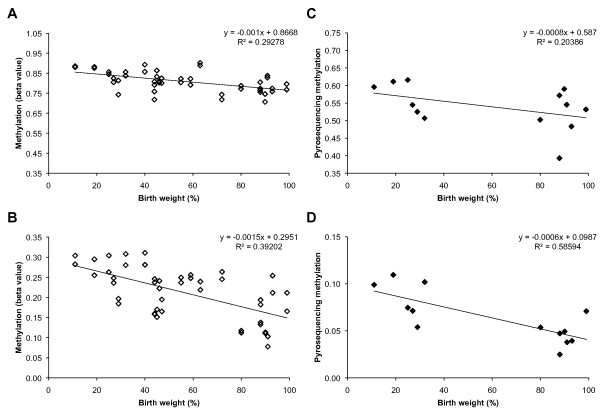
**Methylation levels of candidate genes versus birth weight**. "Beta value" is fraction of methyl C observed at the *PGRMC1 *(CpG ID: cg19606309) site **(A) **and *RGS14 *(CpG ID: cg09010421) site **(B) **on Illumina GoldenGate array in placenta (n = 22, in duplicate). Methylation levels assayed at same site by bisulfite pyrosequencing also correlated with birth weight for *PGRMC1 ***(C) **and *RGS14 ***(D) **in placenta (n = 13).

We validated the array-based methylation levels of these new candidates by bisulfite pyrosequencing of individuals at the highest and lowest ends of the birth weight distribution (Figure 2). Although the absolute levels of methylation measured differ slightly between the two techniques, methylation levels at each validated locus are correlated with birth weight in both cases (Figure 2).

We then tested whether cord blood and placenta methylation levels at these six candidate genes were also correlated with birth weight in the second sample of 48 individuals. Although the individual CpG sites assayed for each gene were not identical between the two arrays, promoter methylation levels at these six candidates were also correlated with birth weight in the second sample of 48 individuals, accounting for 50% of the trait variance (Table 3).

**Table 3:** Candidate genes whose methylation is correlated with birth weight

Data Set	L_1_-regularized regression **R**^**2 1**^	**Non-zero regressions/total "leave one out" regressions at maximum L_1 _R**^**2**^	Tissue	Genes in model	Gene ID
**22 newborns, methylation at 1,536 CpGs assayed using Illumina's GoldenGate array**	0.78	21/22	Blood	*APOE*	Apolipoprotein E
				*MSX1*	Msh homeobox 1
			Placenta	*GRB10*	Growth factor receptor-bound protein 10
				*PGRMC1*	Progesterone receptor membrane component 1
				*RGS14*	Regulator of G-protein signaling 14
				*SHMT2*	Serine hydroxymethyl transferase 2 (mitochondrial)
**48 newborns, methylation at 27,578 CpGs assayed using Illumina's Infinium array**	0.50	44/48	Blood and placenta, as above	Six genes, above	
**48 newborns, methylation at 27,578 CpGs assayed using Illumina's Infinium array**	0.70	45/48	Blood	*ATP6AP1*	Atpase, H + transporting, lysosomal accessory protein 1
				*PRSS21*	Protease, serine, 21 (testisin)
				*RCOR1*	REST co-repressor 1
			Placenta	*ANGPT4*	Angiopoietin 4
				*CDK2*	Cyclin-dependent kinase 2
				*EVPL*	Envoplakin
				*NAT8L*	FLJ37478: N-acetyltransferase 8-like (GCN5-related, putative)
**48 newborns, methylation at 27,578 CpGs assayed using Illumina's Infinium array**	0.84	44/48	Blood and Placenta, as above	All 13 genes from both experiments, combined	

^1 ^The maximum L_1_-regularized regression correlation obtained for the gene model in which more than 90% of the "leave one out" cross-validations exhibited non-zero regression parameters (third column)

**Figure 3:**
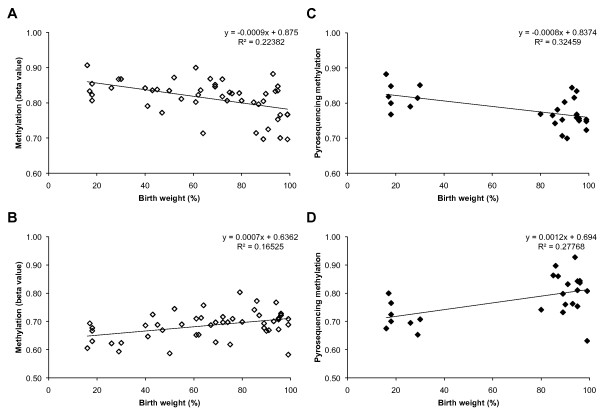
**Methylation levels of candidate genes versus birth weight**. "Beta value" is the fraction of methyl C observed at the *ANGPT4 *(CpG ID: cg26540515) site **(A) **in placenta and *PRSS21 *(CpG ID: cg21085768) site **(B) **in cord blood on the Illumina Infinium array (n = 48). Methylation levels assayed at same site by bisulfite pyrosequencing also correlated with birth weight for *ANGPT4 ***(C) **in placenta (n = 26) and *PRSS21 ***(D) **in cord blood (n = 25).

Although the replication of a correlation between birth weight and methylation level provides a measure of confidence that the candidate genes identified in the training sample of 22 individuals are involved in birth weight, we note that the candidate genes were identified from an original sample of only 1,536 CpGs in 740 loci [32]. In the second sample of 48 individuals, methylation levels were examined at 27,578 CpG sites in 14,495 genes, providing an opportunity to identify birth weight-related methylation differences in many more CpGs/candidate genes. We repeated the L_1_-regularized regression procedure using the larger data set and identified an additional set of seven genes (*ATP6AP1, PRSS21, RCOR1, ANGPT4, CDK2, EVPL *and *NAT8L*), whose methylation levels explained 70% of the variance in birth weight, independently (Table 3). We note that mouse orthologues of two of these genes (*Angpt4 *and *Cdk2*) are associated with growth-related phenotypes [52,53]. *CDK2 *is a central regulator of cell division and *ANGPT4 *is an angiogenesis factor that is expressed in a wide variety of human tissues [54]. Validation of array-based inter-individual methylation differences that correlated with birth weight was performed for selected CpGs by bisulfite pyrosequencing (Figure 3).

The combined model, using methylation levels at all 13 candidate genes identified in both experiments, explains 84% of the variance in birth weight in the sample of 48 individuals (Table 3).

### Transcript levels of candidate genes at delivery are not correlated strongly with birth weight

Transcript levels of 12 of the 13 candidate genes from Table 3 (*NAT8L *is not interrogated by the array), measured at the single time point of delivery, were subject to the L_1 _regression procedure to determine whether methylation levels or transcript levels were better correlated with birth weight. Notably, the single time point transcript levels of these genes do not correlate strongly with birth weight, explaining a maximum of 16% of trait variance (and this maximum correlation is obtained only when the stability of the model is reduced to non-zero regression coefficients in only 42 out of 48 "leave one individual out" validations).

We asked whether the reason that transcript level differences in the 13 candidate genes did not explain variance in birth weight as well as DNA methylation differences was that DNA methylation levels were not correlated with transcript levels of these genes at birth, in *cis*. In fact, only two of the candidates, *EVPL *and *GRB10*, showed significant correlation between methylation of CpG sites at the locus and transcript level, measured at delivery, and only in placenta (Table 4). Interestingly, methylation of CpG sites in *MSX1 *(a homeobox transcriptional repressor) is correlated with transcript level of four of the candidates (Table 5), methylation of CpG sites in *CDK2 *is correlated with transcript level of three of the candidates (Table 5) and methylation of CpG sites in *GRB10 *is correlated with transcript level of four of the candidates (Table 5). In all but two cases, correlations between multiple CpGs in one candidate and transcript level in the other are in the same direction and of similar magnitude (Table 5), suggesting that the effects we observe are not anomalous or limited to single CpG sites but that methylation levels over broad regions of *MSX1, CDK2 *and *GRB10 *(4,272 bp, 372 bp and 12,177 bp, respectively) are correlated with transcript level of the other candidates.

**Table 4:** Correlation between DNA methylation and transcription of candidate genes

Tissue	Methylation Genes	CpG ID	Transcript ID	**Correlation**^**1**^	*P *value
**Placenta**	*EVPL*	cg24697031	ILMN_1727288	-0.30	0.04
	*GRB10*	cg06386517	ILMN_1669617	0.34	0.02
		cg20651681		0.39	0.01
		cg06790324		0.29	0.04
		cg03104936		0.29	0.05
		cg03104936	ILMN_1652662	0.37	0.01
		cg06386517	ILMN_2340919	0.33	0.02
		cg20651681		0.34	0.02
		cg24183958		0.38	0.01
		cg06790324		0.39	0.01

^1 ^Pearson correlation coefficient

**Table 5:** Correlation between DNA methylation and transcription in the candidate genes

Tissue	Methylation Candidate	CpG ID	Gene Transcript	Transcript ID	**Correlation**^**1**^	*P *value
**Blood**	*MSX1*	cg14167596	APOE	ILMN_1740938	0.76	< 0.001
		cg11930592	ATP6AP1	ILMN_1697694	0.32	0.03
		cg15755084			0.32	0.03
		cg20891301^a^			-0.30^2^	0.04
		cg26615830			0.32	0.03
		cg15696627			0.44	0.002
		cg03717979			0.51	< 0.001
		cg15755084	PRSS21	ILMN_2382964	-0.38	0.01
		cg15696627			-0.37	0.01
		cg20588069			-0.42	0.003
		cg06677140			-0.29	0.04
		cg09573795		ILMN_1774256	-0.34	0.02
		cg03199651			-0.30	0.04
		cg20588069			-0.36	0.01
		cg22609784			-0.31	0.03
		cg15696627	RCOR1	ILMN_1743421	-0.28	0.05
		cg03717979		ILMN_1743421	-0.29	0.04
		cg06677140		ILMN_1743421	-0.38	0.01
**Placenta**	*CDK2*	cg09106999	GRB10	ILMN_1667771	-0.32^3^	0.03
		cg00129774		ILMN_1669617	0.51	< 0.001
		cg00129774		ILMN_2340919	0.46	0.001
		cg04108502			0.37	0.01
		cg09304040	PGRMC1	ILMN_1684771	-0.34	0.02
		cg09106999	RGS14	ILMN_1696828	-0.31	0.03
	*EVPL*	cg24697031	EVPL	ILMN_1727288	-0.30	0.04
	*GRB10*	cg20651681	CDK2	ILMN_1653443	-0.29	0.04
		cg15774495			-0.28	0.05
		cg06790324			-0.36	0.01
		cg06386517	GRB10	ILMN_1669617	0.34	0.02
		cg20651681			0.39	0.01
		cg06790324			0.29	0.04
		cg03104936			0.29	0.05
		cg03104936		ILMN_1652662	0.37	0.01
		cg06386517		ILMN_2340919	0.33	0.02
		cg20651681			0.34	0.02
		cg24183958			0.38	0.01
		cg06790324			0.39	0.01
		cg20651681	PGRMC1	ILMN_1684771	-0.30	0.04
		cg03104936			-0.29	0.05
		cg20651681	RGS14	ILMN_1696828	-0.34	0.02
	*NAT8L*	cg08211091	GRB10	ILMN_1669617	-0.31	0.03

^1 ^Pearson correlation coefficient

^2 ^*MSX1 *has five CpGs that are positively correlated with ATP6AP1, however, cg20891301, which is anomalously negatively correlated is located at the end of the CpG island

^3 ^*CDK2 *has three CpGs that are positively correlated with GRB10, however, one CpG, cg09106999 is negatively correlated

We next applied the L_1_-regularized regression procedure to all 48,000 transcripts and identified five candidate genes whose transcript levels are correlated with birth weight (Table 6). These five candidates (only one of which corresponds to an annotated gene) explain 55% of the variance in birth weight, compared with the methylation candidates 70-84% of trait variance explained (Table 3).

**Table 6:** Candidate genes whose transcript levels are correlated with birth weight

Data Set	L_1_-regularized regression **R**^**2**^	**Non-zero regressions/total "leave one out" regressions at maximum L_1 _R**^**2**^	Tissue	Genes in model	Description
**48 newborns, expression at 47,000 transcripts assayed using Illumina's HumanHT-12 v3 Expression BeadChip**	0.55	45/48	Blood	HS.406106	BX090408 Soares fetal liver spleen 1NFLS Homo sapiens cDNA clone IMAGp998E08415; IMAGE:211951
				LOC255130	PREDICTED: Homo sapiens hypothetical LOC255130 (LOC255130)
			Placenta	HS.568324	AGENCOURT_7975600 NIH_MGC_113 Homo sapiens cDNA clone IMAGE:6215286 5
				HS.572889	DA236664 BRAWH3 Homo sapiens cDNA clone BRAWH3033381 5
				NBPF10	Homo sapiens neuroblastoma breakpoint family

### Comparison of the L_1_-regularized regression with "bootstrap" models

The substantial fraction of birth weight trait variance (46-84%) explained by promoter methylation levels at a modest number of genes (between six and 19) is somewhat surprising and caused us to consider the possibility that random collections of similar numbers of genes might perform as well.

As a way of determining the likelihood of obtaining models that explain such a large fraction of variance by chance, we compare the machine learning L_1_-regularized regression procedure with random permutations of six and seven genes to determine what fraction of randomly generated data sets would explain as large or larger a fraction of birth weight variance as the L_1 _procedure. We computed the R^2 ^of each model to generate a distribution of random permutation R^2^'s. The probability of obtaining a model as good or better than the L_1 _model at random is thus the fraction of random permutation models whose R^2 ^equals or exceeds the R^2 ^of each L_1 _model.

We applied the L_1_-regularized regression procedure to 1,000 iterations of random sets of six genes, selected from the 1,536 CpGs in the first methylation array (from which the six gene L_1 _model was derived), and computed their adjusted R^2^. We found that only five of the random models had an adjusted R^2 ^greater than the direct L_1_-regularized regression model (*i.e*., "bootstrapped" significance of the L_1 _model, *P *= 0.005). We then tested each of the five random six-gene models in the second data set to assess what fraction of birth weight variance was explained in an independent experiment. Only two of these six-gene models had positive regression coefficients when applied to the second data set (Adjusted R^2 ^= 0.59, stability 46/48, and R^2 ^= 0.48, stability 44/48, Table 7), indicating that only two of the 1,000 random models generated were robust in explaining birth weight variance.

**Table 7:** Five random permutation models with higher R^2 ^than the L_1 _model and the adjusted R^2 ^when tested on the Infinium data-set

Data Set	**R**^**2**^	Adjusted R^2 ^and stability when tested on Infinium Data	Genes in model	Gene names
**22 newborns, methylation at 1,536 CpGs assayed using Illumina's GoldenGate array**	0.86	0.59 (46/48)	*ADAM9*	ADAM metallopeptidase domain 9
			*DPYSL3*	dihydropyrimidinase-like 3
			*FABP5*	fatty acid binding protein 5
			*HOXB4*	homeobox B4
			*MHC2TA*	CIITA, class II, major histocompatibility complex, transactivator
			*PRO1853*	C2orf56, chromosome 2 open reading frame 56
	0.82	negative	*GRB10*	growth factor receptor-bound protein 10
			*HRASLS3*	PLA2G16, phospholipase A2, group XVI
			*MYH14*	myosin, heavy chain 14, non-muscle
			*NM15555*	
			*WNT16*	wingless-type MMTV integration site family, member 16
	0.81	0.48 (44/48)	*BEST1*	bestrophin 1
			*IMPDH2*	IMP (inosine 5'-monophosphate) dehydrogenase 2
			*OSBPL5*	oxysterol binding protein-like 5
			*PAX3*	paired box 3
			*PSMC3*	proteasome (prosome, macropain) 26S subunit, ATPase, 3
			*SERPINF1*	serpin peptidase inhibitor, clade F (alpha-2 antiplasmin, pigment epithelium derived factor), member 1
	0.80	0 (45/48)	*CBX1*	chromobox homolog 1
			*EOMES*	eomesodermin
			*PABPC4*	poly(A) binding protein, cytoplasmic 4 (inducible form)
			*PIK3CG*	phosphoinositide-3-kinase, catalytic, gamma polypeptide
			*SLC16A1*	solute carrier family 16, member 1 (monocarboxylic acid transporter 1)
			*TMPO*	thymopoietin
	0.79	negative	*C11ORF15*	TMEM9B, TMEM9 domain family, member B
			*CCT3*	chaperonin containing TCP1, subunit 3 (gamma)
			*MYH9*	myosin, heavy chain 9, non-muscle
			*PROX1*	prospero homeobox 1
			*REST*	RE1-silencing transcription factor
			*RPS2*	ribosomal protein S2

We also generated 1,000 random seven-gene models from the 48-sample Infinium data set and computed R^2 ^for each. Twenty-five of these models had adjusted R^2 ^as high or higher than the direct L_1 _seven-gene model ("bootstrapped" significance of the L_1 _model, *P *= 0.025). We then combined the two, six-gene models which also explained variance in the second data set (*i.e*., achieved a positive R^2 ^on the Infinium data) with each of the 25, seven-gene models to create 50, 13-gene models and asked what fraction of these explained as high or higher a fraction of variance as the L_1_, 13-gene model. We found that only one of the 50 resulting models achieved an R^2 ^greater than the L_1 _13-gene model (*i.e*., *P *= 0.02) (Table 8). These data indicate that the L_1_-regularized regression procedure is a valuable method for identifying small groups of genes whose methylation levels are correlated with birth weight and that random groups of genes of the same size perform as well only rarely.

**Table 8:** Each of the two random permutation six-gene models that also had positive R^2 ^in the Infinium data set (from Table 7) were combined with each random permutation seven-gene model that achieved an R^2 ^higher than the L_1 _Infinium model (25 models) for a total of 50, 13 gene models

Data Set	Genes in model	Adjusted R^2 ^and stability when tested on Infinium Data	Genes in resulting model	Gene name
**GoldenGate gene** **model which achieved R^2 ^= 0.48 (stability 44/48) on the Infinium Data**	*BEST1* *IMPDH2*	0.87 (44/48)	*CTTN* *GMDS*	Cortactin GDP-mannose 4,6-dehydratase
	***OSBPL5***		*IMPDH2*	IMP (inosine 5'-monophosphate) dehydrogenase 2
	***PAX3***		***OSBPL5***	oxysterol binding protein-like 5
	*PSMC3*		**PAX3**	paired box 3
	*SERPINF1*		*PSMC3*	proteasome (prosome, macropain) 26S subunit, ATPase, 3
**Infinium gene model** **which achieved better** **R^2 ^than our model**	*CTTN*		***REG1B***	regenerating islet-derived 1 beta
	*GMDS*		*RUVBL1*	RuvB-like 1 (E. coli)
	***REG1B***		*SERPINF1*	serpin peptidase inhibitor, clade F (alpha-2 antiplasmin, pigment epithelium derived factor), member 1
	*VPS52*		*VPS52*	vacuolar protein sorting 52 homolog
	*RUVBL1*			
	*KIAA241*			

Only one of the 50 models achieved higher R^2 ^than the L_1 _13 gene model. Genes in bold have suggested role in fetal or placental growth and development

The random permutation model that explained the highest fraction of birth weight trait variance combined the six-gene GoldenGate model (*BEST1, IMPDH2, OSBPL5, PAX3, PSMC3 *and *SERPINF1*) with the seven-gene Infinium model (*CTTN, GMDS, REG1B, VPS52, RUVBL1 *and *KIAA241*). When the L_1 _procedure is applied to the 13 genes in the combined model, irrelevant features are eliminated and the resulting model contains only 10 relevant genes (*CTTN, GMDS, IMPDH2, OSBPL5, PAX3, PSMC3, REG1B, RUVBL1, SERPINF1 *and *VPS52*). This 10-gene model achieved an adjusted R^2 ^of 0.87 and three (*OSBPL5, PAX3 *and *REG1B*) of the 10 genes are likely to have a role in growth-related phenotypes.

## Conclusions

### DNA methylation differences may serve as a record of differences in "potential" transcript level or transcript level integrated over time

We have used three approaches to identify genes whose DNA methylation levels or transcript levels may explain a significant fraction of trait variance in individual birth weight. In the first approach, we analyzed 19 genes identified as growth- or birth weight-associated in the literature. We found that although transcript levels of none of the 19 candidates explained very much of the trait variance individually, the 19 candidates, in aggregate, explained 24% of trait variance. Interestingly, promoter DNA methylation levels of these genes explained as much (26% in the first data set) or more (46% in the second data set) of trait variance than did transcript levels.

In the second approach, we used a machine-learning technique (L_1 _regularized regression) to identify genes whose methylation level explained a significant fraction of birth weight trait variance. L_1 _regularized regression selects CpG sites whose methylation levels are correlated with birth weight and tests whether the association is robust by performing multiple "leave one sample out" tests of whether the correlation remains. Genes with consistent correlations are kept and added to the model and irrelevant genes are discarded. The contribution of each gene is then evaluated individually and in combination with the other candidates until additional features no longer make a significant impact on the adjusted R^2^. This procedure identified six genes whose methylation levels explained 78% of birth weight trait variance. Only two of the six genes, *APOE *and *GRB10*, have been identified previously as associated with growth phenotypes. However, the contribution of these six genes to birth weight appears robust because they explained 50% of the variance in an independent data set and explained an equal or greater fraction of birth weight trait variance in both data sets than did the 19 mechanism-based candidate genes (78% *vs*. 26% and 50% *vs*. 46%) tested in the first approach. We also used the L_1 _regression approach to identify candidate genes from amongst the much larger number of candidates evaluated in the second data set and identified seven genes, of which only two (*ANGPT4 *and *CDK2*) were associated previously with growth. The combination of all 13 L_1 _candidate genes gave an adjusted R^2 ^of 0.84 in the larger data set, indicating that this method of identifying genes that affect birth weight is superior to the mechanism-based candidate gene approach.

Because DNA methylation levels of this small number of genes unexpectedly explained such a large fraction of trait variance, we added a third approach and compared the efficacy of random collections of six and seven genes to explain a similar fraction of trait variance. We found that only two of 1,000 six gene models (*P *= 0.002), 25 of 1,000 seven gene models (*P *= 0.025) and only one of the 50 resulting combined models (*P *= 0.02) performed as well as the L_1 _model. From a computational standpoint, the L_1 _method has substantial advantages over the random permutation method (beginning with the uncertainty of how many genes to sample at a time in the random permutation/bootstrap method) and is likely to become even more valuable when larger data sets involving more individuals and more irrelevant features (larger CpG arrays) become available.

It is noteworthy that none of the transcript level-based models did as well in explaining birth weight trait variance as the corresponding methylation level-based models (Tables 3, 7, 8). This circumstance suggests that the candidate genes exert their largest effect on fetal or placental growth cumulatively or at some period prior to delivery. While this assertion is not surprising, it suggests, further, that inter-individual differences in candidate gene DNA methylation may serve as a kind of "fossil record" of candidate gene expression differences during development. Such inter-individual differences that track birth weight *via *the DNA methylation of candidate loci may be less likely to change dramatically over the course of development than transcript levels that are dependent largely on the action of factors that act in *trans *[55,56].

A major question posed by the data in Tables 3, 7 and 8 concerns the fact that the best models share no genes in common. This circumstance suggests that very little precision or predictive ability is to be gained by increasing the number of genes in a model beyond six - 13. While this conclusion does not imply that only a very small number of genes are involved in controlling birth weight, it does suggest that methylation levels of genes in one model are correlated with methylation of genes in the other models such that any of a suite of correlated genes will predict birth weight as well as any of the others in the same suite. The fact that each model contains genes that have been demonstrated to affect growth in functional studies provides some assurance that the genes identified are actually affecting birth weight in a significant way. Even if many genes contribute incrementally to growth, our analysis indicates that relatively few explain a large enough fraction of variance that they will be identified by examining small populations.

### Potential roles of the candidate genes in determining birth weight

Overall, we have identified 23 genes whose methylation levels are correlated strongly with birth weight. In addition to the four genes known to affect growth in the 13 gene L_1 _model (*APOE, GRB10, ANGPT4 *and *CDK2*), several of the genes identified in the random permutation model are likely to be involved in weight regulation and/or appear to play a role in growth and development. Oxysterol binding protein-like 5 (*OSBPL5*), an imprinted gene with preferential expression from the maternal allele (only in placenta), plays a key role in the maintenance of cholesterol balance in the body. Fatty acid binding protein 5 (*FABP5*) plays a role in fatty acid uptake, transport and metabolism and polymorphisms in this gene are associated with type 2 diabetes. Furthermore, mice homozygous for disruptions in this gene display resistance to diet-induced obesity (depending on the allele), showing decreased adipose tissue and improved glucose tolerance and insulin sensitivity. The protein encoded by the homeobox B4 (*HOXB4*) gene functions as a sequence-specific transcription factor that is involved in development, and the transcription factor paired box 3 (*PAX3*) may play a critical role during fetal development. Regenerating islet-derived 1 beta (*REG1B*) encodes a protein secreted by the exocrine pancreas that is highly similar to the *REG1A *protein, which is associated with islet cell regeneration and diabetogenesis, and may be involved in pancreatic litho genesis. Mice homozygous for a null allele also exhibit impaired suckling.

### Potential confounders of the role of candidate gene methylation in determining birth weight

There are two sources of error that could influence the results of our analysis and diminish the strength of the associations observed. The first is error in assigning the correct birth weight to any individual child. Birth weight is a complex phenotype, influenced by gestational age, maternal weight and age, parity, infant sex and race [57,58], as well as other factors. Although our sample (Additional file 1) has small numbers of non-Caucasian infants, we have adjusted birth weight percentile considering only gestational age. While it is possible that consideration of these multiple additional confounders would alter slightly the placement of individual babies in the birth weight distribution, it is also possible that such adjustments would be performed erroneously. For example, the major objection to including non-Caucasian infants in the analysis is likely to be that Asian and African American infants are smaller than Caucasian infants. However, the two African American children in our sample are at the 89^th ^and 96^th ^birth weight percentile and the one fully Asian child is at the 80^th ^percentile (Additional file 1). We decided to use the single most important contributor to birth weight (gestational age) as our only adjustment to the primary phenotype to avoid the potential for multiple confounder adjustment to categorize phenotype erroneously.

The second source of error that would reduce the reproducibility of the model is the potential for assigning methylation levels incorrectly. This could happen as a result of intra-individual variation in methylation levels because of placental tissue mosaicism or variation in subpopulations of cord blood lymphocytes. Although such variation does have the potential to result in mis-assigning methylation levels, the actual influence of these variations is likely to be small, in practice. Even though flow-sorted subpopulations of lymphocytes may show significant gene-specific variation in methylation levels (*e.g*., B cells *vs*. CD4 T-cells *vs*. CD8 T-cells in Figure 1 in Rakyan et al. 2008) longitudinal measures of site-specific DNA methylation in total lymphocytes taken from the same individuals, decades apart, rarely change by more than a few percent [59-61]. We have also examined the effect of inflammatory markers (erythrocyte sedimentation rate and levels of C-reactive protein) likely to be associated with specific leukocyte subpopulations, as well as total white blood cell count in longitudinal studies of 111 individuals [61] and none of these parameters was related to any methylation differences observed [61]. Similarly, in terms of placental subpopulations, we have compared DNA methylation levels at the *IGF2*/*H19 *and *IGF2R *DMRs in five section of placenta both within and between individuals. Although there is some variation within a placenta, there is substantially more variation between individuals than within an individual [27]. These observations suggest that intra-individual variation in placental or cord blood DNA methylation are unlikely to change the correlations observed between candidate gene methylation and birth weight.

### Candidate gene interaction may identify novel regulatory networks and provide links between low birth weight and adult disease

Of the birth weight-associated candidate gene DNA methylation differences identified in the L_1 _procedure (Table 3), three are of particular interest. Methylation levels of the homeobox transcriptional repressor *MSX1 *in cord blood are correlated with the transcript level of four of the other candidate genes (*APOE, ATP6AP1, PRSS21 *and *RCOR1 *(Table 5)). In fact, at least seven of the top 10 genes whose transcript level is correlated with methylation of CpG sites in *MSX1 *(Table 9) are suspected to play roles in fetal or placental growth. On the placental side, methylation levels of multiple sites in *CDK2 *are correlated with expression of three of the other candidates and multiple CpG sites in *CDK2 *are correlated with transcript levels of *GRB10 *(Table 5). Methylation levels of multiple sites in *GRB10 *are correlated with transcript levels of four of the seven candidates (*CDK2, GRB10, PGRMC1 *and *RGS14*), including itself (Table 5), and two of the genes in the top ten *GRB10 *transcript level correlations (Table 9) have been found to have an effect on growth.

**Table 9:** Top ten genes whose transcript levels are correlated with methylation of CpG sites in *MSX1, CDK2 *and *GRB10*

Tissue	Methylation Gene	CpG ID	Expression Gene	Transcript ID	**Correlation**^**1**^	Gene Name
**Blood**	***MSX1***	cg14167596	**APOE**	ILMN_1740938	0.76	Apolipoprotein E
		cg14167596	**CGA**	ILMN_1734176	0.70	Glycoprotein Hormones, Alpha Polypeptide
		cg03199651	KRT6C	ILMN_1754576	0.69	Keratin 6 C
		cg14167596	PAPPA	ILMN_1721770	0.67	Protein Kinase C And Casein Kinase Substrate in Neurons 1
		cg14167596	**PSG4**	ILMN_1693397	0.67	Pregnancy Specific Beta-1-Glycoprotein 4
		cg26615830	DCN	ILMN_2347145	0.65	Decorin
		cg14167596	**PSG6**	ILMN_2309615	0.65	Pregnancy Specific Beta-1-Glycoprotein 6
		cg14167596	**CSH1**	ILMN_1693617	0.65	Chorionic somatomammotropin hormone 1 (placental lactogen)
		cg14167596	**GH2**	ILMN_1659354	0.65	Growth Hormone 2
		cg14167596	**ADAM12**	ILMN_1726266	0.65	ADAM Metallopeptidase Domain 12
**Placenta**	***CDK2***	cg04108502	CXCL11	ILMN_2067890	0.74	Chemokine (C-X-C Motif) Ligand 11
		cg04108502	HLA-DPB1	ILMN_1749070	0.70	Major Histocompatibility Complex, Classii, DP Beta 1
		cg04108502	CXCL9	ILMN_1745356	0.68	Chemokine (C-X-C Motif) Ligand 9
		cg04108502	GBP4	ILMN_1771385	0.68	Guanylate Binding Protein 4
		cg04108502	GBP5	ILMN_2114568	0.67	Guanylate Binding Protein 5
		cg04108502	UBD	ILMN_1678841	0.66	Ubiquitin D
		cg04108502	VCY	ILMN_1683872	0.66	Variable charge, Y-linked
		cg04108502	HLA-DRB3	ILMN_1717261	0.66	Major Histocompatibility Complex, Class II, DR Beta 3
		cg04108502	CD3D	ILMN_2261416	0.65	CD3d molecule, delta (CD3-TCR complex)
		cg04108502	CETP	ILMN_1681882	0.65	Cholesteryl ester transfer protein, plasma
	***GRB10***	cg20651681	SHROOM2	ILMN_1681777	0.68	Shroom Family Member 2
		cg20651681	**MESDC1**	ILMN_1781565	0.67	Mesoderm Development Candidate 1
		cg20651681	CCDC146	ILMN_1790555	0.67	Coiled-Coil Domain Containing 146
		cg20651681	VANGL2	ILMN_1715647	0.67	Vang-Like2 (Vangogh, Drosophila)
		cg06790324	SCG2	ILMN_1703178	0.66	Secretogranin II
		cg20651681	STGC3	ILMN_1807244	0.66	hypothetical STGC3
		cg06790324	ABHD14B	ILMN_2227533	0.66	Ab Hydrolase Domain Containing 14B
		cg20651681	**TLL1**	ILMN_1699814	0.66	Tolloid-Like 1
		cg20651681	SOD1	ILMN_1662438	-0.65	Superoxide Dismutase 1, Soluble
		cg06790324	INPP5E	ILMN_1811301	0.65	Inositol polyphosphate-5-phosphatase, 72 kDa

Genes in bold have suggested role in fetal or placental growth and development

^1 ^Pearson correlation coefficient (*P *≤ 0.01)

The mechanisms linking low birth weight to adverse long-term health outcomes are not well understood but may be related to defective placentation, restrictions in the size of stem cell populations that lead to reduced organ size and function, and/or abnormal programming of metabolic pathways including glucose utilization. In this regard, it is noteworthy that methylation levels of three CpGs in the *MSX1 *transcriptional repressor are correlated with transcript levels of the glucose transporter *SLC2A3 *(Pearson correlation coefficient 0.42).

Furthermore, *GRB10 *methylation is also correlated with expression of genes involved in reactive oxygen species (ROS) signaling, stress signaling and oxygen sensing. This is of interest because *GRB10 *is transcriptionally imprinted in human villous trophoblasts (and brain) and proliferation/differentiation of trophoblast cells is responsive to oxygen tension [62-64]. *GRB10 *has known major effects on placental growth. More recent data implicate *GRB10 *in insulin signaling [65,66], which suggests a mechanism and pathway by which a neonatal phenotype could be linked to adult disease. Discovery of such "unexpected" pathways may inform about the long-term association between low birth weight and adult disease, as well as which genes may be susceptible to environmental effects.

The association we have identified between candidate gene methylation levels (at birth) and birth weight suggests that methylation levels of the candidates do not change significantly during early development. Although we have not documented that the methylation states of the candidate genes do not change during development, we have shown previously that fewer than 10% of individuals exhibit global methylation changes of more than 20% when measured longitudinally, over decades [61]. We also demonstrated that only 21 genes, of 805 examined (2.6%), showed methylation changes of greater than 20% over the same period [61]; *i.e*., approximately 1% change per year. The fact that these gene-specific changes were observed in individuals from a single family with the greatest difference in global methylation [66] between the two sampling times suggests that large changes in DNA methylation levels over time are relatively uncommon. Given such temporal stability, it may be possible to understand how inter-individual epigenetic differences, observed at birth, predispose some individuals to undesirable outcomes later in life.

## Competing interests

The authors declare that they have no competing interests.

## Authors' contributions

NT carried out the biochemical and molecular analyses, participated in the data analyses and drafted the manuscript. SK carried out the gene expression assays. MFG carried out the bioinformatic and statistical analyses and helped to draft the manuscript. ZO, CC and CS conceived of the study, participated in its design and coordination and helped to draft the manuscript, which was initially written by CS. All authors read and approved the final manuscript.

## Pre-publication history

The pre-publication history for this paper can be accessed here:

http://www.biomedcentral.com/1755-8794/5/10/prepub

## Supplementary Material

Additional file 1 **Demographic data for subjects in the GoldenGate and Infinium Methylation Assays. Birth weights were corrected for gestational age **[57,58,67]. Click here for file
