# Supplementary material for: DNA methylation differences at growth related genes correlate with birth weight: a molecular signature linked to developmental origins of adult disease?
Source: BMC Med Genomics. 2012 Apr 12;5:10. doi: 10.1186/1755-8794-5-10 (PMC3359247; doi:10.1186/1755-8794-5-10)
Supplement: Additional file 1 — Demographic data for subjects in the GoldenGate and Infinium Methylation Assays. Birth weights were corrected for gestational age [57,58,67]. [file 1755-8794-5-10-S1.PDF]

Additional file 1. Demographic data for subjects in the GoldenGate and Infinium Methylation Assays. Birth weights were corrected for gestational age [57, 58, 67].

Assay	Maternal Age (yrs)	Race Ethnicity (M)	Race Ethnicity (P)	Parity (FT)	Sex	Birth Weight (g)	Gestational Age (wks)	Birth Weight (%)
**GoldenGate Methylation Assay**	36	Hispanic	White	1	M	3742	37 5/7	83
	35	White	White	1	M	2039	32 2/7	47
	35	White	Hispanic	0	F	3827	40 4/7	72
	35	White	White	0	F	1626	31 6/7	19
	39	White	White	0	M	1360	29 5/7	32
	37	White	White	0	M	3458	39 5/7	45
	33	White	White	0	F	3660	39 6/7	63
	40	White	White	0	F	2779	38 2/7	11
	40	White	White	0	M	3232	38 2/7	44
	35	White	White	3	M	4020	38 3/7	93
	23	White	White	0	M	3856	38 3/7	88
	36	White	White	3	F	3315	38 4/7	40
	38	White	White	2	M	3972	39	88
	34	White	White	1	F	4528	38 5/7	99
	30	White	White	0	M	3474	39	55
	33	White	White	0	M	3794	39 3/7	80
	31	White	White	0	M	3356	38 5/7	44
	40	Hispanic	White	0	M	3523	41	46
	33	White	White	2	M	3662	41 2/7	59
	31	White	White	0	F	3260	40 4/7	25
	32	White	White	1	F	3796	37	91
	27	White	White	0	M	3043	38 1/7	27
**Infinium Methylation Assay**	36	Hispanic	White	1	M	3742	37 5/7	90
	32	Asian	White	0	F	2892	37 1/7	30
	35	White	White	1	F	4100	39 2/7	93
	35	White	Hispanic	0	F	3827	40 4/7	72
	37	White	White	0	M	3317	41 3/7	29
	33	White	White	0	M	4447	40 5/7	96
	37	White	White	0	M	3458	39 5/7	45
	34	White	White	3	F	3402	40 1/7	40
	39	White	White	0	M	3416	39 6/7	41
	40	White	White	4	M	3714	39 3/7	74
	42	White	White	0	M	3667	40 2/7	75
	34	White	Hispanic	2	M	3570	38 1/7	72
	36	White	White	0	M	4318	41 5/7	95
	34	African American	African American	0	F	3985	38 4/7	89
	45	White	White	1	F	3741	38 6/7	76
	25	White	White	0	F	3125	39 5/7	18
	41	White	White	0	F	4130	39 6/7	91
	38	White	White	0	M	3696	40 6/7	61
	41	White	White	0	M	3008	38 5/7	16
	30	White	White	0	F	3660	39 6/7	63
	29	White	White	0	F	4174	39 3/7	95
	39	White	White	1	M	4659	39 2/7	99
	38	Asian	White	0	F	3668	41 4/7	62
	40	White	White	0	F	3520	38	69
	38	White	White	1	M	3033	39	18
	34	White	White	1	F	4528	38 5/7	99
	40	Hispanic	White	0	M	3523	41	47
	32	White	White	0	M	3535	40	52
	32	Hispanic	White	0	M	3948	39 2/7	87
	43	White	White	3	M	3225	38	43
	30	White	White	0	F	3230	39 5/7	26
	28	White	White	1	F	3740	39 4/7	69
	31	White	White	1	M	4620	38	99
	37	White	White	1	F	4200	39	95
	36	White	White	2	M	3892	39	85
	39	African American	African American	3	F	4290	39	96
	35	White	White	1	F	4070	40	89
	33	Asian	Asian	0	F	3528	37	80
	30	White	White	0	M	3120	40 1/7	18
	38	White	White	0	F	3145	40 5/7	17

31	White	White	0	F	3940	41 3/7	79
31	White	White	1	M	4150	39 3/7	94
28	White	White	4	M	3690	41 1/7	61
35	White	Asian	1	F	3565	40	55
38	White	White	2	M	4000	39 4/7	86
41	White	White	0	M	3620	39 3/7	67
32	White	White	1	M	3510	39 5/7	50
31	White	White	0	F	3584	38 4/7	64
